# ﻿*Mutatus* gen. nov., a new genus of Phrurolithidae (Arachnida, Araneae) from China, with descriptions of four new species

**DOI:** 10.3897/zookeys.1226.137752

**Published:** 2025-02-06

**Authors:** Yannan Mu, Luyu Wang, Feng Zhang, Zhisheng Zhang

**Affiliations:** 1 Key Laboratory of Eco-environments in Three Gorges Reservoir Region (Ministry of Education), School of Life Sciences, Southwest University, Chongqing 400715, China Hebei University Baoding China; 2 Key Laboratory of Zoological Systematics and Application, College of Life Sciences, Hebei University, Baoding, Hebei 071002, China Southwest University Chongqing China

**Keywords:** Asia, guardstone spiders, morphology, taxonomy

## Abstract

*Mutatus***gen. nov.** is described from southwest China, including four new species: *M.gelao***sp. nov.** (♂♀, Guizhou Province), *M.tianyan***sp. nov.** (♂♀, Hubei Province), *M.tujia***sp. nov.** (♂♀, Hubei Province), and *M.yintiaoling***sp. nov.** (♂, Chongqing Municipality). This new genus can be differentiated from other phrurolithid genera by the male palp which has a prolateral tegular apophysis and tegular sheet, and by a small protuberance on the female epigynal plate.

## ﻿Introduction

The family Phrurolithidae Banks, 1892 comprises 405 species in 25 genera and is mainly distributed in the Holarctic and Southeast Asia, but with a few species known from Africa and Australia. Members of the family Phrurolithidae usually inhabit leaf litter on the forest floor, the undersides of stones and tree bark (Liu et al. 2019), or the walls of abandoned houses (Mu et al. 2022). Currently, 17 genera and 213 species have been reported from China (WSC 2024), of which about 14 genera and 128 species have been documented within the past five years (Liu et al. 2020, 2022; Kamura 2021; Mu and Zhang 2021, 2022, 2023; Mu et al. 2022). These studies have significantly advanced the taxonomy of phrurolithid spiders.

While examining phrurolithid specimens collected in Chongqing Municipality, and Hubei and Guizhou provinces, we identified species exhibiting a unique combination of traits. The presence of a tegular sheet suggested a close relationship with *Lingulatus* Mu & Zhang, 2022, while the prolateral tegular apophysis, indicates an affinity with *Xilithus* Liu & Li, 2023. Based on this unique combination of characteristics, we describe four new species as members of a new genus, *Mutatus* gen. nov.

## ﻿Materials and methods

All specimens are preserved in 95% alcohol. All measurements in the text are given in millimeters. The measurements of legs are shown as total length (femur, patella, tibia, metatarsus, tarsus). The palps were dissected from the femur. The epigyna were removed and cleared in a pancreatin solution (Álvarez-Padilla and Hormiga 2007) and then transferred to 95% ethanol. Photographs were taken using the Leica M205A stereomicroscope equipped with a DFC550 CCD. All specimens are deposited in the Museum of Hebei University (**MHBU**), Baoding, China.

The following abbreviations are used: **AER**—anterior eye row; **ALE**—anterior lateral eye; **AME**—anterior median eye; **CH**—clypeal height; **CRW**—cephalic region width; **CT**—connecting tube; **CW**—carapace width; **EAW**—eye area width; **FA**—femur apophysis; **MOA**—median ocular area; **MS**—median septum; **PLE**—posterior lateral eye; **PME**—posterior median eye; **PER**—posterior eye row; PTA—prolateral tegular apophysis; **RTA**—retrolateral tibial apophysis; **TS**—tegular sheet; **RvTA**—retroventral tibial apophysis. Spination: **d**—dorsal; **pl**—prolateral; **pv**—proventral; **rv**—retroventral.

## ﻿Taxonomy

**Phrurolithidae Banks, 1892**

### ﻿ Mutatus gen. nov.

Taxon classificationAnimaliaAraneaePhrurolithidae

4F3F5182-DED7-567F-80EC-075F0E84E938

https://zoobank.org/5B7451D2-FC3D-4564-9391-EE277E0EC926

#### Etymology.

The genus name is derived from a Latin word “*mutatio*”, meaning “variable”, referring to the unique combination of characters in this new genus. Unlike the similar genera *Lingulatus*, which possesses only a tegular sheet, and *Xilithus*, which features only prolateral tegular apophysis, the new genus exhibits both characteristics in the male palp. Gender masculine.

#### Diagnosis.

The male of new genus resembles those of the genera *Lingulatus* and *Xilithus* in having both tegular sheet and prolateral tegular apophysis, but *Mutatus* can be differentiated by the absence of a ventral tibial apophysis (present both in *Lingulatus* and *Xilithus*), tegular sheet present in both *Mutatus* gen. nov. and *Lingulatus* (absent in *Xilithus*), and sperm duct V-shaped in both *Mutatus* gen. nov. and *Lingulatus* (U-shaped in *Xilithus*). The female of new genus resembles *Xilithus* in having a pair of large concavities (atria), a pair of small bursae, and clavate spermathecae, but it can be differentiated by the presence of a protuberance at the middle part of the lateral margin of the atrium (absent in *Xilithus*).

#### Description.

Small, total length 3.18–4.78 mm. Carapace oval, smooth, brownish to black, widest at coxae II and III, highest near fovea, with several markings resembling flowing water droplets beside fovea. Fovea dark-red. PER slightly wider than AER, PER recurved in dorsal view, AER recurved in dorsal view. Chelicerae brown, with 1 long and 1 short spine anteriorly. Endites longer than wide, labium wider than long. Sternum brown, shield-shaped, smooth and without pattern, longer than wide. Legs dark brown, all femora with 1 dorsal spine; femur I with 3–5 prolateral spines distally, femur II with 0–2 prolateral spines distally; tibiae I–II usually with 6–7 pairs of ventral spines, tibiae III–IV without ventral spines; metatarsus I with 4–5 pairs of ventral spines, metatarsus II usually with 4 proventral spines and 3 retroventral spines, metatarsi III–IV without ventral spines but with distal preening brush. Leg formula 4123. Abdomen oval, gray to black in dorsal view, with dorsal scutum covering almost entire abdomen in males, scutum in females absent.

Male palp: femur with well-developed apophysis located at middle part; patella short, as long as tibia; tibia slightly bulged ventrally, with retroventral and retrolateral apophyses; cymbium longer than femur; bulb pyriform; prolateral tegular apophysis and tegular sheet (spade-shaped) originating near base of embolus; embolus acicular; conductor absent.

Epigyne: epigynal plate weakly sclerotized, with pair of atria; septum dumbbell-shaped; copulatory openings small, separated; copulatory ducts thin; connecting tubes thick, shorter than copulatory ducts; bursae small; spermathecae clavate, located posteriorly; glandular appendages mastoid-shaped.

#### Type species.

*Mutatusgelao* sp. nov.

#### Composition.

*Mutatusgelao* sp. nov., *M.tujia* sp. nov., *M.tianyan* sp. nov., and *M.yintiaoling* sp. nov.

#### Distribution.

China (Hubei Province, Guizhou Province, Chongqing Municipality).

### ﻿ Mutatus gelao sp. nov.

Taxon classificationAnimaliaAraneaePhrurolithidae

CA91E2E8-A5E2-5AFF-A948-2937CE5FBB61

https://zoobank.org/C66AD91F-662D-45DE-9698-204FD511F7BD

**Figure 1.**
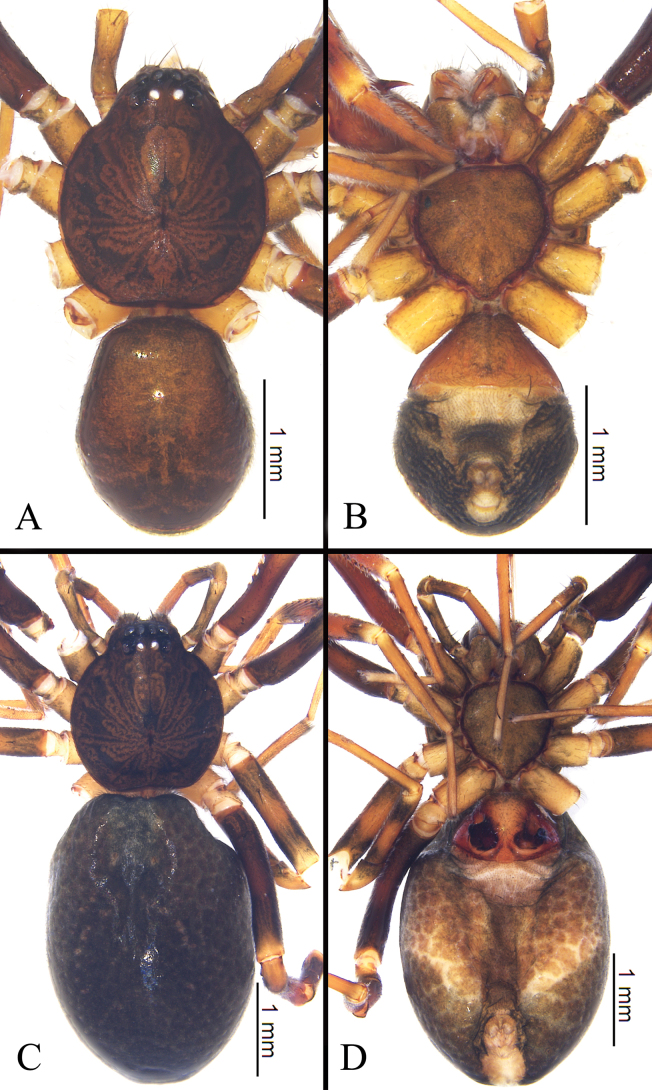
Habitus of *Mutatusgelao* sp. nov. **A** male holotype, dorsal view **B** same, ventral view **C** female paratype, dorsal view **D** same, ventral view.

**Figure 2.**
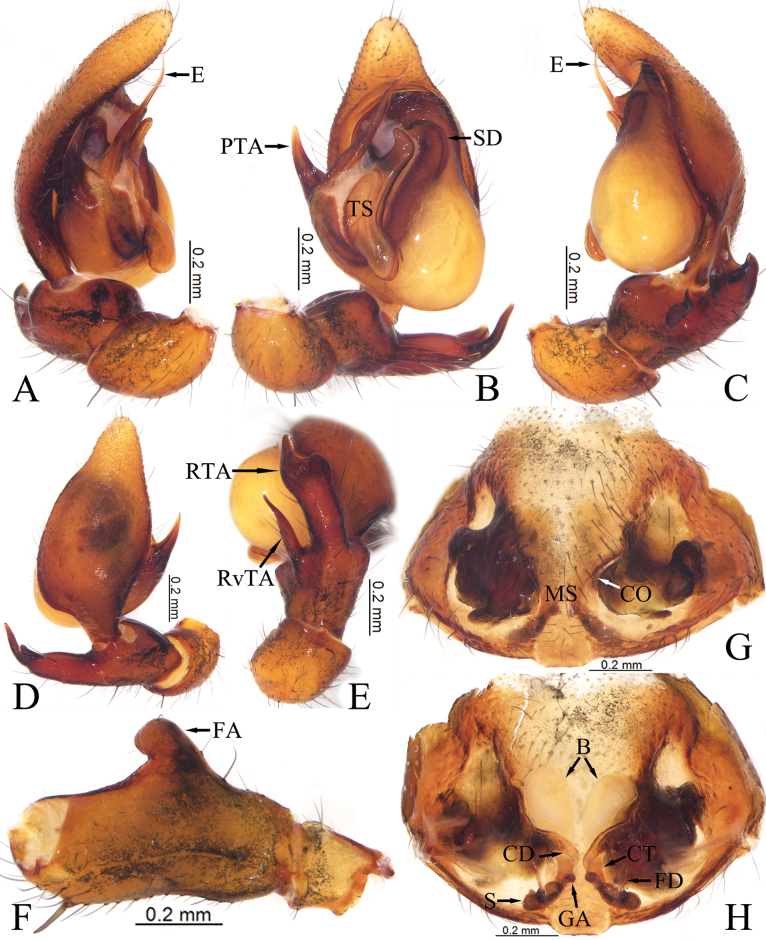
Copulatory organs of *Mutatusgelao* sp. nov. **A** male left palp, prolateral view **B** same, ventral view **C** same, retrolateral view **D** same, dorsal view **E**RTA, retrolateral view **F** femur, prolateral view **G** epigyne, ventral view **H** same, dorsal view. Abbreviations: E—embolus; FA—femoral apophysis; PTA—prolateral tegular apophysis; RTA—retrolateral tibial apophysis; RvTA—retroventral tibial apophysis; SD—sperm duct; TS—tegular sheet; B—bursa; CD—copulatory duct; CO—copulatory opening; CT—connecting tube; FD—fertilization duct; GA—glandular appendage; MS—median septum; S—spermathecae.

Chinese name: 仡佬幻蛛 Figs 1 , 2

#### Type material.

***Holotype***: China • ♂; Guizhou Province, Tongren City, Shiqian County, Gelao Village; 27°2'3.12"N, 108°9'6.94"E, elev. 647 m, 08 May 2023; Z.Y. Li, W.H. Wang leg. ***Paratype***: 1♀, with same data as holotype.

#### Etymology.

The specific name comes from the word “Gelao”. The name is one of the Chinese ethnic minorities that live adjacent to the area inhabited by the new species; a noun in apposition.

#### Diagnosis.

The new species can be differentiated from *Mutatustujia* sp. nov. and *M.tianyan* sp. nov. by the ventrally bulging tibia of the male palp (vs not inflated; compare Fig. 2C with Figs 4B, 6B), almost bifurcate tip of the retrolateral tibial apophysis (vs smooth and tapering from base to tip; compare Fig. 2C with Figs 4C, 6D), spade-shaped tegular sheet (vs tobacco-pipe-shaped, compare Fig. 2B with Figs 4D, 6C), spiculate prolateral tegular apophysis (vs truncated, compare Fig. 2B with Figs 4D, 6C), copulatory ducts which form a right angle with connecting tubes (vs forming a obtuse angle; compare Fig. 2H with Figs 4F, 6F), and larger and deeper, kidney-shaped atria (vs atria oval, small, and shallow; compare Fig. 2G with Figs 4E, 6E).

#### Description.

Male (holotype, Fig. 1A, B). Total length 3.35, carapace 1.73 long, 1.55 wide; abdomen 1.62 long, 1.32 wide. Eye sizes and interdistances: AME 0.11, ALE 0.13, PME 0.09, PLE 0.10; AME–AME 0.04, AME–ALE 0.01, ALE–ALE 0.27, PME–PME 0.10, PME–PLE 0.08, PLE–PLE 0.41, ALE–PLE 0.09. EAW 0.56, CRW 0.81, EAW/CRW 0.69. CRW/CW 0.52. MOA 0.27 long, anterior width 0.24, posterior width 0.26. Clypeal height 0.15, CH/AME 1.36. Labium 0.22 long, 0.24 wide. Sternum 0.96 long, 0.96 wide. Carapace oval, cervical groove inconspicuous. Sternum, labium, and endite brown. Abdomen dark brown, smooth, with large dorsal scutum covering almost entire abdomen. Legs brown.

Palp (Fig. 2A–F). Femoral apophysis (FA) well developed, thumb-shaped, located at middle part. Patella about 1/3 length of femur, as long as tibia. Tibia longer than wide, slightly bulged ventrally, retroventral apophysis (RvTA) tail-shaped, tip slightly curved; retrolateral apophysis (RTA) longer and wider than RvTA, tip bifurcated. Bulb pyriform, sperm duct (SD) V-shaped. Prolateral tegular apophysis (PTA) coniform, slightly curved, originating at nearly 9 o’clock position. Tegular sheet (TS) spade-shaped, anterior part with curved tip, posterior part tongue-shaped. Embolus (E) needle-like, tapering towards tip, slightly curved.

Female (Fig. 1C, D). Total length 4.78, carapace 1.79 long, 1.64 wide; abdomen 3.08 long, 2.27 wide. Eye sizes and interdistances: AME 0.11, ALE 0.12, PME 0.08, PLE 0.10; AME–AME 0.04, AME–ALE 0.01, ALE–ALE 0.24, PME–PME 0.08, PME–PLE 0.08, PLE–PLE 0.39, ALE–PLE 0.09. EAW 0.54, CRW 0.80, EAW/CRW 0.68. CRW/CW 0.49. MOA 0.28 long, anterior width 0.24, posterior width 0.24. Clypeal height 0.16, CH/AME 1.36. Labium 0.23 long, 0.25 wide. Sternum 1.07 long, 0.97 wide. Other characters as for male, except dorsal scutum absent, color darker and body slightly larger.

Epigyne (Fig. 2G, H). Epigynal plate slightly sclerotized, with a pair of atria. Median septum (MS) dumbbell-shaped. Copulatory openings (CO) small, located at anterior of atrium. Copulatory ducts (CD) slightly curved, X-shaped, longer and thinner than connecting tubes (CT). Bursae (B) nearly balloon-shaped, translucent. Glandular appendages (GA) mastoid-shaped. Spermathecae (S) clavate, separated by septum. Fertilization ducts (FD) located at median margin of spermathecae.

Measurement of legs:

**Table:** 

male/female	Fe	Pa	Ti	Me	Ta	Total
Leg I	1.67/1.71	0.60/0.56	1.80/1.65	1.58/1.51	0.89/0.85	6.54/6.27
Leg II	1.54/1.63	0.47/0.61	1.44/1.49	1.20/1.38	0.86/0.93	5.51/6.04
Leg III	1.31/1.43	0.51/0.54	1.07/1.07	1.22/1.33	0.70/0.77	4.81/5.14
Leg IV	1.96/2.10	0.58/0.65	1.79/1.73	2.01/2.06	1.02/0.97	7.36/7.51

Spination of legs:

**Table:** 

		Fe	Pa	Ti	Me	Ta
male	Leg I	d 1 pl 4	–	pv 7 rv 7	pv 4 rv 4	–
	Leg II	d 1 pl 0	–	pv 7 rv 5	pv 4 rv 4	–
	Leg III	d 1	–	–	–	–
	Leg IV	d 1	–	–	–	–
female	Leg I	d 1 pl 5	–	pv 7 rv 7	pv 5 rv 5	–
	Leg II	d 1 pl 2	–	pv 7 rv 7	pv 4 rv 3	–
	Leg III	d 1	–	–	–	–
	Leg IV	d 1	–	–	–	–

#### Distribution.

Known only from the type locality.

### ﻿ Mutatus tujia sp. nov.

Taxon classificationAnimaliaAraneaePhrurolithidae

2374E035-C29E-54C0-A042-3255513F03F7

https://zoobank.org/995C189B-E908-4132-BD2C-5F62D4BB9794

**Figure 3.**
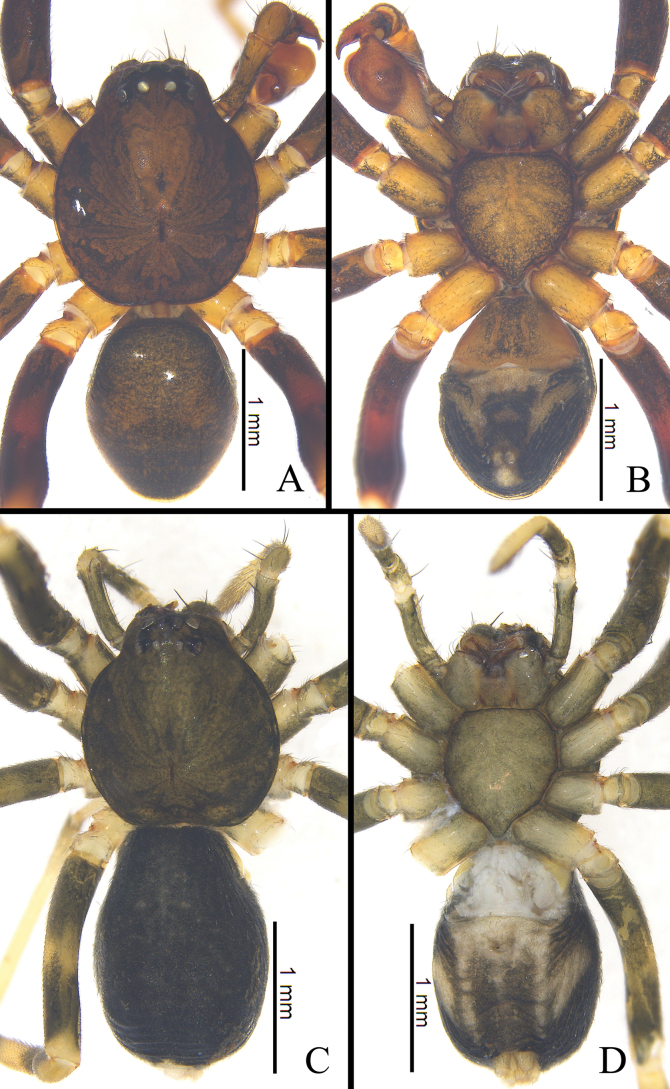
Habitus of *Mutatustujia* sp. nov. **A** male holotype, dorsal view **B** same, ventral view **C** female paratype, dorsal view **D** same, ventral view.

**Figure 4.**
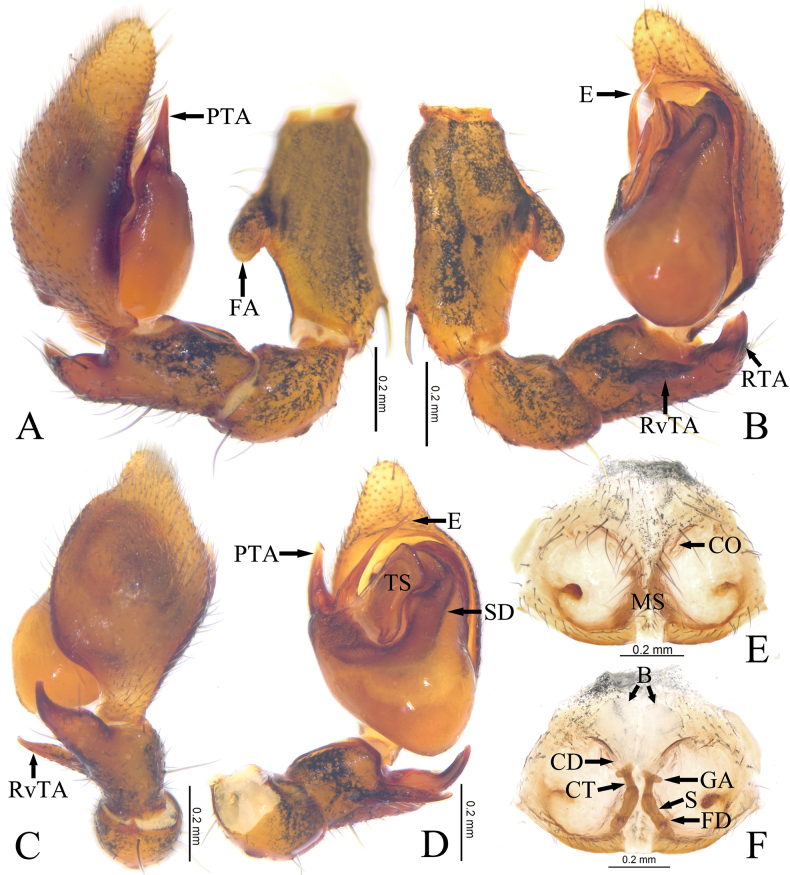
Copulatory organs of *Mutatustujia* sp. nov. **A** male left palp, prolateral view **B** same, retrolateral view **C** same, dorsal view **D** same, ventral view **E** epigyne, ventral view **F** same, dorsal view. Abbreviations: E—embolus; FA—femoral apophysis; PTA—prolateral tegular apophysis; RTA—retrolateral tibial apophysis; RvTA—retroventral tibial apophysis; SD—sperm duct; TS—tegular sheet; B—bursa; CD—copulatory duct; CO—copulatory opening; CT—connecting tube; FD—fertilization duct; GA—glandular appendage; MS—median septum; S—spermathecae.

Chinese name: 土家幻蛛 Figs 3 , 4 , 8

#### Type material.

***Holotype***: China • ♂; Hubei province, Shennongjia Forest District, Xiaguping, Tujiazu Township, Huangxi River; 31°21'59.87"N, 110°11'18.32"E, elev. 1059 m, 19 May 2023; Z.Y. Li, Z.Y. Yang leg. ***Paratypes***: • 1♂ 7♀, with same data as holotype; • 2♂ 4♀ same locality, Dongkou Village (31°22'54.25"N, 110°9'48.53"E, 1053 m a.s.l.), 19 May 2023, Z.Y. Li, Z.Y. Yang leg.

#### Etymology.

The specific name comes from the word “Tujia”. The name is one of the Chinese ethnic minorities that live adjacent to the area inhabited by the new species; a noun in apposition.

#### Diagnosis.

The new species resembles *M.tianyan* sp. nov. in having similar truncated prolateral tegular apophysis and embolus, but it can be differentiated by the following: posterior of tegular sheet tail-shaped (vs tongue-shaped; compare Fig. 4D with Fig. 6C); median septum thin (vs thick; compare Fig. 4E with Fig. 6E); bursa large, balloon-shaped (vs small, rectangular-shaped; compare Fig. 4F with Fig. 6F); glandular appendages distinct, large (vs indistinct, small; compare Fig. 4F with Fig. 6F).

#### Description.

Male (holotype, Fig. 3 A, B). Total length 3.34, carapace 1.85 long, 1.61 wide; abdomen 1.35 long, 1.22 wide. Eye sizes and interdistances: AME 0.09, ALE 0.13, PME 0.07, PLE 0.08; AME–AME 0.06, AME–ALE 0.02, ALE–ALE 0.27, PME–PME 0.11, PME–PLE 0.07, PLE–PLE 0.45, ALE–PLE 0.10. EAW 0.58, CRW 0.83, EAW/CRW 0.70, CRW/CW 0.52. MOA 0.25 long, anterior width 0.22, posterior width 0.29. CH 0.11, CH/AME 1.22. Labium 0.35 long, 0.30 wide. Sternum 1.21 long, 1.17 wide. Carapace oval, cervical groove inconspicuous. Sternum, labium, and endite brown, without pattern. Abdomen dark brown, smooth, with large dorsal scutum covering almost entire abdomen. Legs brown.

Palp (Fig. 4A–D). Femoral apophysis (FA) well developed, thumb-shaped, located at middle part. Patella as long as tibia, about 1/3 length of femur. Tibia longer than wide, with 2 apophyses, retroventral apophysis (RvTA) tail-shaped; retrolateral apophysis (RTA) strong, longer and wider than retroventral apophysis, tapering towards tip. Bulb pyriform, sperm duct (SD) distinct, V-shaped. Prolateral tegular apophysis (PTA) coniform, slightly curved, originating at nearly 9 o’clock position. Tegular sheet (TS) tobacco pipe-shaped, anterior part large and wide, posterior part small, tail-shaped. Embolus (E) needle-like, tapering towards tip, slightly curved.

Female (one of the paratypes; Fig. 3C, D). Total length 3.50, carapace 1.66 long, 1.49 wide; abdomen 1.86 long, 1.25 wide. Eye sizes and interdistances: AME 0.08, ALE 0.09, PME 0.07, PLE 0.08; AME–AME 0.06, AME–ALE 0.02, ALE–ALE 0.23, PME–PME 0.10, PME–PLE 0.06, PLE–PLE 0.35, ALE–PLE 0.09. EAW 0.48, CRW 0.71, EAW/CRW 0.68, CRW/CW 0.47. MOA 0.23 long, anterior width 0.23, posterior width 0.18. CH 0.10, CH/AME 1.25. Labium 0.23 long, 0.20 wide. Sternum 0.87 long, 0.75 wide. Other characters as for male, except dorsal scutum absent, color black and body slightly larger.

Epigyne (Fig. 4E, F). Epigynal plate slightly sclerotized, with a pair of atria, a subcircular tumor located at middle part of lateral margin of atrium. Median septum (MS) dumbbell-shaped. Copulatory openings (CO) slit-like, located at anterior of atrium. Copulatory ducts (CD) straight, short, indistinct. Connecting tubes (CT) distinct. Bursae (B) large, nearly balloon-shaped, translucent. Glandular appendages (GA) mastoid-shaped. Spermathecae (S) clavate, anterior part close but separated posteriorly. Fertilization (FD) ducts located at posterior end of spermathecae.

Measurement of legs:

**Table:** 

male/female	Fe	Pa	Ti	Me	Ta	Total
Leg I	1.38/1.44	0.43/0.46	1.42/1.58	1.14/1.34	0.61/0.69	4.98/5.51
Leg II	1.20/1.19	0.45/0.44	1.10/1.21	0.96/1.07	0.58/0.58	4.29/4.40
Leg III	1.00/1.01	0.39/0.39	0.80/0.81	0.91/1.01	0.57/0.58	3.67/3.80
Leg IV	1.50/1.62	0.44/0.46	1.32/1.53	1.45/1.60	0.77/0.89	5.48/6.10

Spination of legs:

**Table:** 

		Fe	Pa	Ti	Me	Ta
male	Leg I	d 1 pl 3	–	pv 7 rv 7	pv 4 rv 4	–
	Leg II	d 1 pl 0	–	pv 6 rv 6	pv 4 rv 3	–
	Leg III	d 1	–	–	–	–
	Leg IV	d 1	–	–	–	–
female	Leg I	d 1 pl 3	–	pv 6 rv 6	pv 4 rv 4	–
	Leg II	d 1 pl 0	–	pv 6 rv 6	pv 4 rv 4	–
	Leg III	d 1	–	–	–	–
	Leg IV	d 1	–	–	–	–

#### Distribution.

Known only from the type locality.

### ﻿ Mutatus tianyan sp. nov.

Taxon classificationAnimaliaAraneaePhrurolithidae

5B085250-E23D-5468-B9FF-2085D7785F85

https://zoobank.org/AC6ABF0C-10FD-4FF0-A548-5FDE4EF2EA4A

**Figure 5.**
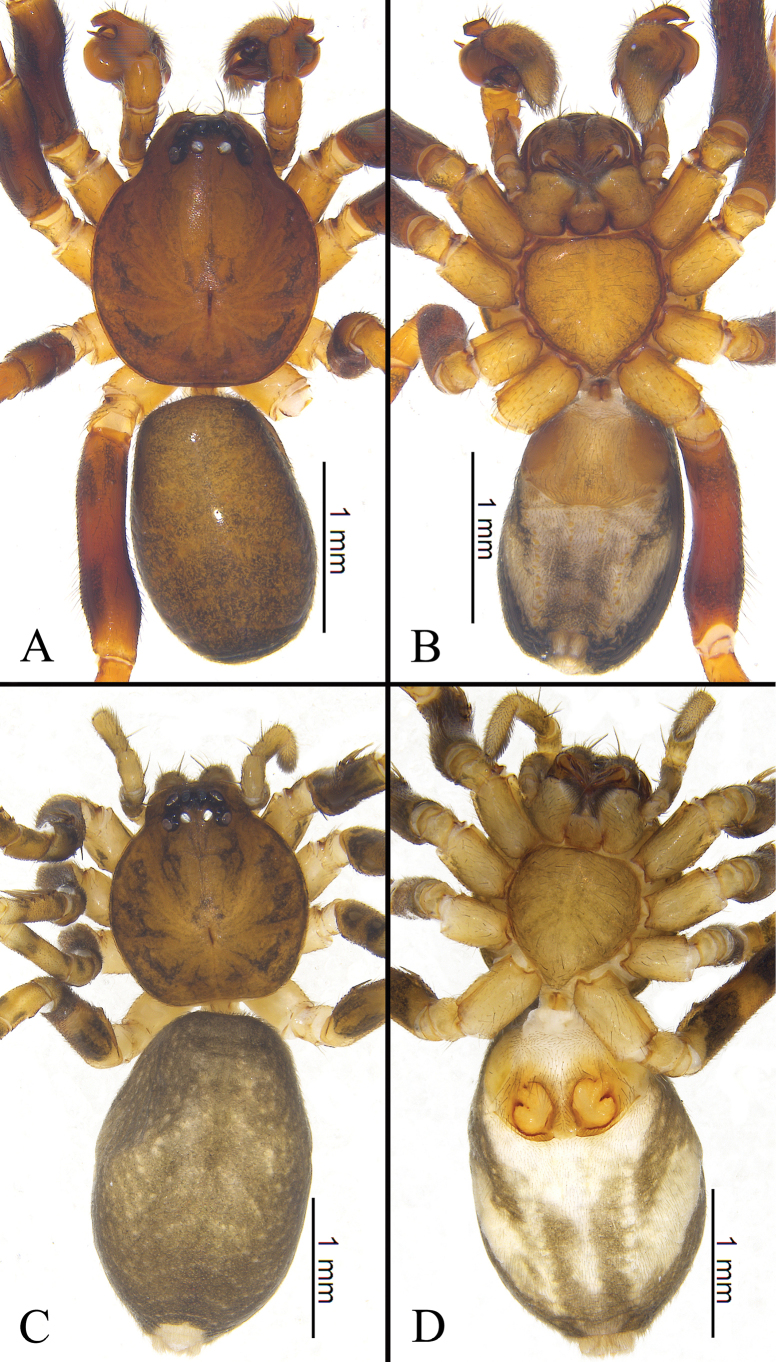
Habitus of *Mutatustianyan* sp. nov. **A** male holotype, dorsal view **B** same, ventral view **C** female paratype, dorsal view **D** same, ventral view.

**Figure 6.**
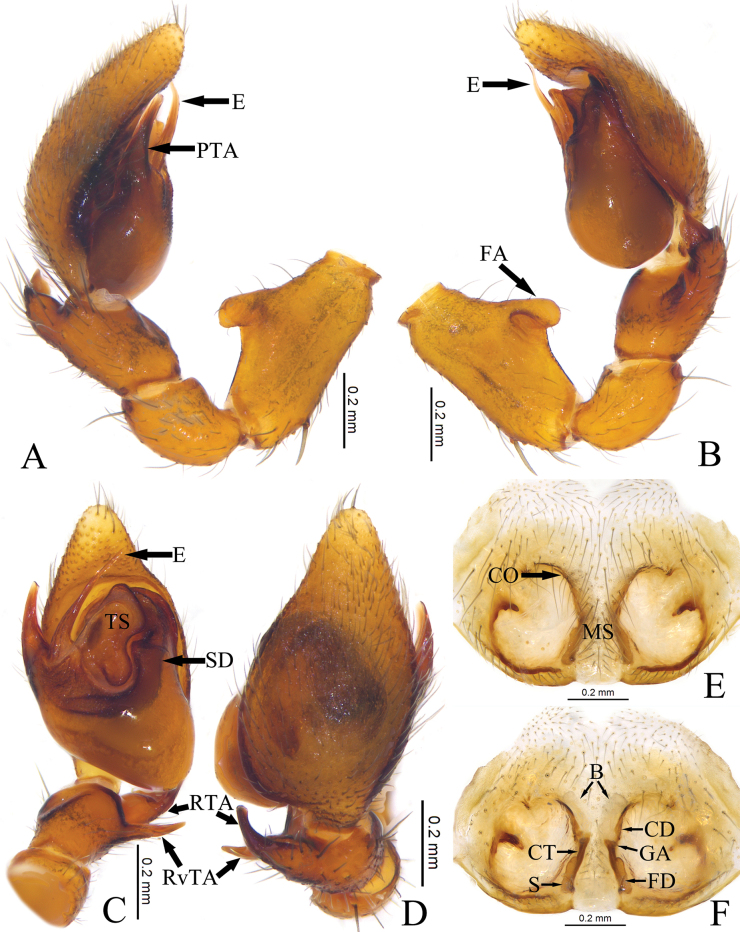
Copulatory organs of *Mutatustianyan* sp. nov. **A** male left palp, prolateral view **B** same, retrolateral view **C** same, ventral view **D** same, dorsal view **E** epigyne, ventral view **F** same, dorsal view. Abbreviations: E—embolus; FA—femoral apophysis; PTA—prolateral tegular apophysis; RTA—retrolateral tibial apophysis; RvTA—retroventral tibial apophysis; SD—sperm duct; TS—tegular sheet; B—bursa; CD—copulatory duct; CO—copulatory opening; CT—connecting tube; FD—fertilization duct; GA—glandular appendage; MS—median septum; S—spermathecae.

Chinese name: 天燕幻蛛 Figs 5 , 6 , 8

#### Type material.

***Holotype***: China • ♂ Hubei province: Shennongjia Forest District, Tianyan Primitive Ecotourism Area; 31°43'16"N, 110°28'31"E, elev. 1871 m, 14 June 2023, Z.S. Zhang, Y.J. Cai leg. ***Paratype***: • 7♀, with same data as holotype.

#### Etymology.

This specific name is derived from the type locality; a noun in apposition.

#### Diagnosis.

See the diagnosis of *M.tujia* sp. nov.

#### Description.

Male (holotype, Fig. 5A, B). Total length 3.18, carapace 1.59 long, 1.35 wide; abdomen 1.59 long, 1.05 wide. Eye sizes and interdistances: AME 0.07, ALE 0.09, PME 0.08, PLE 0.09; AME–AME 0.06, AME–ALE 0.02, ALE–ALE 0.24, PME–PME 0.08, PME–PLE 0.08, PLE–PLE 0.40, ALE–PLE 0.06. EAW 0.52, CRW 0.74, EAW/CRW 0.70. CRW/CW 0.55. MOA 0.22 long, anterior width 0.19, posterior width 0.25. Clypeal height 0.15, CH/AME 2.14. Labium 0.21 long, 0.25 wide. Sternum 0.96 long, 0.91 wide. Carapace oval, cervical groove inconspicuous. Sternum, labium, and endite brown, without pattern. Abdomen brown, smooth, with large dorsal scutum covering almost entire abdomen. Legs brown.

Palp (Fig. 6A–D). Femoral apophysis (FA) well developed, thumb-shaped, located at middle part. Patella longer than tibia, as half the length of femur. Tibia longer than wide, with 2 apophyses, retroventral apophysis (RvTA) tail-shaped, tapering towards tip; retrolateral apophysis (RTA) strong, longer and wider than retroventral apophysis, tip curved ventrally in dorsal view. Bulb pyriform, sperm duct (SD) distinct, V-shaped. Prolateral tegular apophysis (PTA) originating at nearly 9 o’clock position, slightly curved, tip truncated. Tegular sheet (TS) spade-shaped, anterior part large and wide, posterior part small, tongue-shaped. Embolus (E) needle-like, tapering towards tip.

Female (one of paratype, Fig. 5C, D). Total length 4.02, carapace 1.55 long, 1.44 wide; abdomen 2.47 long, 1.56 wide. Eye sizes and interdistances: AME 0.07, ALE 0.10, PME 0.07, PLE 0.10; AME–AME 0.05, AME–ALE 0.02, ALE–ALE 0.24, PME–PME 0.08, PME–PLE 0.06, PLE–PLE 0.37, ALE–PLE 0.06. EAW 0.52, CRW 0.75, EAW/CRW 0.69. CRW/CW 0.52. MOA 0.22 long, anterior width 0.19, posterior width 0.25. Clypeal height 0.14, CH/AME 2.00. Labium 0.21 long, 0.25 wide. Sternum 0.96 long, 0.91 wide. Other characters as for male, except dorsal scutum absent, color lighter and body slightly larger.

Epigyne (Fig. 6E, F). Epigynal plate slightly sclerotized, with a pair of atria, a clavate protuberance located at middle part of lateral margin of atrium. Median septum (MS) dumbbell-shaped. Copulatory openings (CO) slit-like, inconspicuous, located at anterior of atrium. Copulatory ducts (CD) straight, indistinct. Connecting tubes (CT) shorter than copulatory ducts. Bursae (B) small, almost rectangular, translucent. Glandular appendages (GA) mastoid-shaped. Spermathecae (S) clavate, separated by median septum. Fertilization ducts (FD) located at posterior of spermathecae.

Measurement of legs:

**Table:** 

male/female	Fe	Pa	Ti	Me	Ta	Total
Leg I	1.49/1.43	0.53/0.57	1.45/1.55	1.30/1.37	0.73/0.77	5.50/5.69
Leg II	1.28/1.27	0.49/0.54	1.09/1.13	1.07/1.10	0.70/0.75	4.63/4.79
Leg III	1.10/1.10	0.46/0.43	0.84/0.82	1.02/1.10	0.61/0.68	4.03/4.13
Leg IV	1.59/1.68	0.53/0.57	1.34/1.45	1.64/1.69	0.84/0.93	5.94/6.32

Spination of legs:

**Table:** 

		Fe	Pa	Ti	Me	Ta
male	Leg I	d 1 pl 4	–	pv 6 rv 6	pv 4 rv 4	–
	Leg II	d 1 pl 1	–	pv 6 rv 4	pv 4 rv 3	–
	Leg III	d 1	–	–	–	–
	Leg IV	d 1	–	–	–	–
female	Leg I	d 1 pl 3	–	pv 7 rv 7	pv 4 rv 4	–
	Leg II	d 1 pl 2	–	pv 6 rv 6	pv 4 rv 3	–
	Leg III	d 1	–	–	–	–
	Leg IV	d 1	–	–	–	–

#### Distribution.

Known only from the type locality.

### ﻿ Mutatus yintiaoling sp. nov.

Taxon classificationAnimaliaAraneaePhrurolithidae

26430641-35E3-57AC-BD98-4644659ADEAB

https://zoobank.org/942E6A33-B2A8-4B4E-9A5C-9EFBEB2379AE

**Figure 7.**
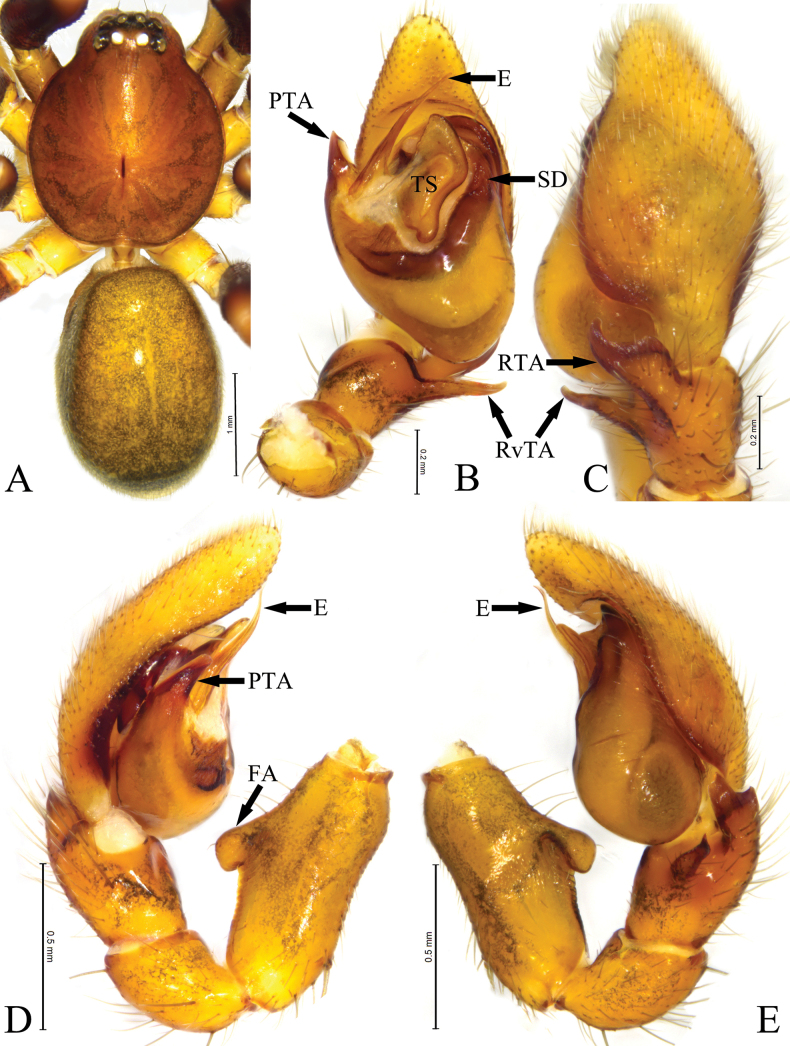
Habitus and copulatory organs of *Mutatusyintiaoling* sp. nov. **A** male holotype, dorsal view **B** male left palp, ventral view **C** same, dorsal view **D** same, prolateral view **E** same, retrolateral view. Abbreviations: E—embolus; FA—femoral apophysis; PTA—prolateral tegular apophysis; RTA—retrolateral tibial apophysis; RvTA—retroventral tibial apophysis; SD—sperm duct; TS—tegular sheet.

**Figure 8.**
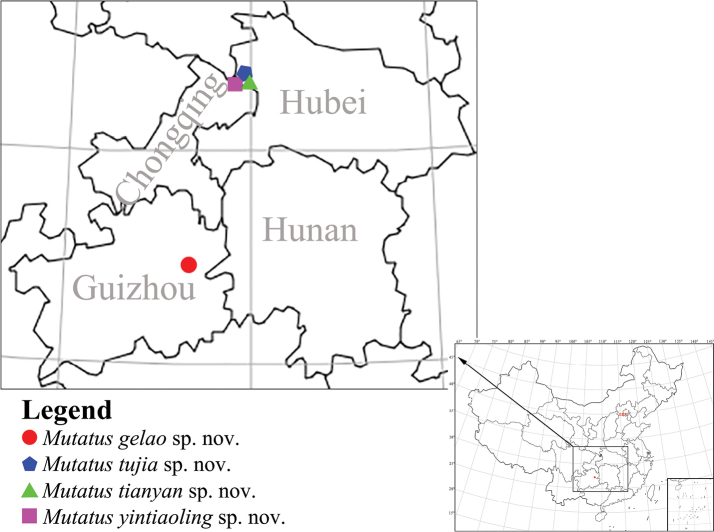
Distribution map of *Mutatus* gen. nov.

Chinese name: 阴条岭幻蛛 Figs 7 , 8

#### Type material.

***Holotype*** ♂ China • Chongqing Municipality: Yintiaoling National Nature Reserve, Zhuanping (31°29'43.12"N, 109°55'17.96"E, elev. 1752 m), 23 May 2022, H.L. Zhu leg.

#### Etymology.

This specific name is derived from the type locality; a noun in apposition.

#### Diagnosis.

This new species can be differentiated from *M.gelao* sp. nov., *M.tujia* sp. nov., and *M.tianyan* sp. nov. by the shorter prolateral tegular apophysis (vs long; compare Fig. 7B with Figs 2B, 4D, 5C) and the truncated and more curved tip of the long branch of retrolateral tibial apophysis (vs not truncated and curved tip; compare Fig. 7C, E with Figs 2E, 4D, 5B).

#### Description.

Male (holotype, Fig. 7A). Total length 3.90, carapace 1.90 long, 1.63 wide; abdomen 2.00 long, 1.28 wide. Eye sizes and interdistances: AME 0.10, ALE 0.13, PME 0.10, PLE 0.10; AME–AME 0.04, AME–ALE 0.02, ALE–ALE 0.27, PME–PME 0.10, PME–PLE 0.06, PLE–PLE 0.42, ALE–PLE 0.07. EAW 0.58, CRW 0.89, EAW/CRW 0.65. CRW/CW 0.54. MOA 0.26 long, anterior width 0.24, posterior width 0.30. Clypeal height 0.15, CH/AME 1.50. Labium 0.25 long, 0.27 wide. Sternum 0.96 long, 1.00 wide. Carapace oval, brown, cervical groove inconspicuous. Sternum brown, without pattern. Labium and endite as same color as sternum. Abdomen lighter-colored than carapace, smooth, with large dorsal scutum covering almost entire abdomen. Legs brown.

Palp (Fig. 7B–E). Femoral apophysis (FA) well developed, located at middle part. Patella longer than tibia, as half the length as femur. Tibia longer than wide, with 2 apophyses, retroventral apophysis (RvTA) tail-shaped, tip slightly curved, tapering towards tip; retrolateral apophysis (RTA) strong, longer and wider than RvTA, abruptly narrowed at middle part in dorsal view, tip curved towards cymbium in dorsal view. Bulb pyriform, sperm duct (SD) distinct, V-shaped. Prolateral tegular apophysis (PTA) short, originating at nearly 9 o’clock position, tip truncated. Tegular sheet (TS) spade-shaped, anterior part large and wide, posterior part small, protuberance-shaped. Embolus (E) needle-like, slightly curved, tapering towards tip.

Female: unknown.

Measurement of legs:

**Table:** 

male	Fe	Pa	Ti	Me	Ta	Total
Leg I	1.62	0.62	1.61	1.60	0.74	6.19
Leg II	1.41	0.61	1.21	1.11	0.73	5.07
Leg III	1.26	0.52	0.94	1.19	0.76	4.67
Leg IV	1.81	0.67	1.41	1.64	0.86	6.39

Spination of legs:

**Table:** 

		Fe	Pa	Ti	Me	Ta
male	Leg I	d 1 pl 3	–	pv 6 rv 6	pv 4 rv 3	–
	Leg II	d 1 pl 2	–	pv 6 rv 5	pv 4 rv 3	–
	Leg III	d 1	–	–	–	–
	Leg IV	d 1	–	–	–	–

#### Distribution.

Known only from the type locality.

## ﻿Discussion

*Mutatus* gen. nov., resembles *Lingulatus* and *Xilithus* in having tegular sheet and prolateral tegular apophysis on the male palp, respectively. In *Lingulatus*, the position of the tegular sheet seems variable and aligns with the position of embolus, which suggests a functional relationship between the tegular sheet and the embolus. In *Mutatus* gen. nov., the position of the tegular sheet and embolus are fixed and resembling the embolus and distal tegular apophysis in *Xilithus*. The distal tegular apophysis appears morphologically matched with the atria in *Xilithus*, while significant differences exist between tegular sheet and atria in *Mutatus* gen. nov. Additionally, the presence of a prolateral tegular apophysis and similar epigynal features further indicate a close relationship between *Mutatus* gen. nov. and *Xilithus*.

If the tegular sheet in *Lingulatus* and *Mutatus* gen. nov. and distal tegular apophysis in *Xilithus* were considered homologous, it could suggest that *Mutatus* gen. nov. and *Lingulatus* are subgroups of *Xilithus*. However, substantial differences between the *Lingulatus* and *Xilithus* argue against this hypothesis. These differences include the presence of a prolateral tegular apophysis and atria in *Xilithus* (absent/occasional in *Lingulatus*), as well as ventral tibial apophysis, tegular sheet, and large bursae found in *Lingulatus* (small or absent in *Xilithus*). Furthermore, the length and spatial path of sperm duct differs significantly between *Lingulatus* and *Xilithus*. So, we believe that the tegular sheet and distal tegular apophysis are not homologous, and the establishment of *Mutatus* gen. nov. as a valid genus is well supported.

## Supplementary Material

XML Treatment for Mutatus

XML Treatment for Mutatus gelao

XML Treatment for Mutatus tujia

XML Treatment for Mutatus tianyan

XML Treatment for Mutatus yintiaoling

## ﻿Additional information

### Author ORCIDs

Yannan Mu https://orcid.org/0000-0002-2504-673X

Luyu Wang https://orcid.org/0000-0002-5250-3473

Feng Zhang https://orcid.org/0000-0002-3347-1031

Zhisheng Zhang https://orcid.org/0000-0002-9304-1789

### Data availability

All of the data that support the findings of this study are available in the main text.
